# The combination therapy of isomucronulatol 7-O-beta-glucoside (IMG) and CEP-9722 targeting ferroptosis-related biomarkers in non-small cell lung cancer (NSCLC)

**DOI:** 10.1186/s12890-023-02445-0

**Published:** 2023-05-11

**Authors:** Xiaofei Cui, Chang Liu, Penghua Dong, Chao Liu, Yu Bai

**Affiliations:** 1 Department of EICU, the Second Affiliated Hospital of Dalian Medical University, Dalian, 116027 Liaoning China; 2 Department of Thoracic Surgery, Shenyang Tenth People’s Hospital, Shenyang Chest Hospital, Shenyang, 110044 Liaoning China; 3 Dalian Medical University, Dalian, Liaoning China; 4 Department of Thoracic Surgery, the Second Affiliated Hospital of Dalian Medical University, Dalian, 116027 Liaoning China

**Keywords:** NSCLC, Ferroptosis, Prognosis, Single-cell sequencing, Integrated traditional and western medicine, Synergy effect

## Abstract

**Background:**

NSCLC is a malignant tumor with a high incidence. Ferroptosis presents an essential function in regulating carcinogenesis and tumor progression. However, the ferroptosis-associated prognostic model based on single-cell sequencing of NSCLC remains unexplored. Our study aims to establish a potential predictive model for NSCLC patients and provide available targeted drugs for clinical treatment.

**Methods:**

The data on NSCLC patients were collected from TCGA and GEO databases to analyze their gene expression profiles. ConsensusCluster was adopted to divide the patients into different groups based on ferroptosis-related genes. Then, the univariable Cox and LASSO analyses were applied to data analysis and model establishment. Single-cell analysis was used to explore the risk score genes in different cell populations and states. The protein levels of these genes were also investigated through the HPA database. Drug sensitivity was evaluated in CellMiner database. CCK8 and colony formation assays were performed to validate potential drugs’ effects on lung cancer cell lines.

**Results:**

A ferroptosis-related prognostic model involving 14 genes in NSCLC patients was established. The risk score model was developed in training set GSE31210 and validated in the test set TCGA. The low-risk score group showed a better prognosis than the high-risk score group. The single-cell analysis revealed that the risk score genes were mainly derived from lung tumor cells. Most risk score genes were more highly expressed in tumor tissue than in normal tissue, according to the HPA database. Besides, these genes were associated with 106 drugs in CellMiner database. Finally, the drug effects on NSCLC cell growth were evaluated by cck8 and colony formation.

**Conclusions:**

We identified an effective ferroptosis-related prognostic model based on single-cell sequencing. The potential prediction model is devoted to exploring clinical therapeutic targets for NSCLC.

**Supplementary Information:**

The online version contains supplementary material available at 10.1186/s12890-023-02445-0.

## Background

Lung cancer is the most common tumor worldwide. The median age of lung cancer patients is 71 years, with approximately 90% of patients older than 55 [1]. Moreover, most patients evolve into advanced stage at diagnosis. Thus, the prognostic outcomes for advanced lung cancer patients are unsatisfactory due to the decreased immunity [2]. In the clinicopathological staging of lung cancer, the proportion of NSCLC has been as high as 85% [3]. According to statistics, only about 15% of NSCLC patients survive longer than 5 years, and the prognosis of patients with advanced-stage is disappointing [4]. In the past, the usual treatment modality for advanced patients was platinum-based doublet chemotherapy [5]. However, most advanced NCSLC patients remain resistant to the current treatment [6, 7]. Therefore, the research on the mechanism related to NSCLC still needs to be further explored.

Cell death is the ultimate fate of cells. The two crucial mechanisms of cell death are accidental cell death (ACD) and regulated cell death (RCD) [8]. RCD is often considered a defense against cancer. Unlike autophagy, apoptosis, and cell necrosis, one of the regulated cell death modalities, ferroptosis is unique cell death form [9]. Cancer cells have defects in some normal executive mechanisms, which is a major reason why cancer cells become resistant to treatment [10]. The growth of cancer cells exhibits a greater iron requirement than normal cells [11]. Therefore, ferroptosis is often considered an adaptive feature in eliminating malignancy and play an important role in tumor suppression [11]. Previous studies also showed that many tumor suppressors regulate ferroptosis and tumors by affecting tumor metabolisms, such as BAP1 and P53 [10, 12, 13]. Thus, the treatment of NSCLC by inducing ferroptosis in cancer cells holds great promise for research. It has been demonstrated that in NSCLC, ginkgetin may promote DDP-induced anticancer effects via ferroptosis induction [14]. However, it remains a challenge that ferroptosis is applied to diagnosis and therapy in cancer research. Therefore, developing new targets and predictive models for cancer treatment is necessary.

CEP-9722 is an inhibitor of PARP1 and PARP2, a prodrug of CEP-8983 [15]. CEP-9722 has been shown to inhibit cell growth in ovarian cancer, colon cancer, glioma, and urothelial cancer [15–18]. However, the role of CEP-9722 in NSCLC has not been investigated. Scutellaria baicalensis Georgi belongs to the Lamiaceae family and is a flowering plant. The root of Scutellaria baicalensis Georgi is used as an herbal remedy for influenza, pneumonia, dysentery, and cancer. Its Chinese name is Hedysarum Multijugum Maxim (HMM) [19, 20]. Recent studies showed that some ingredients of HMM could be used to treat NSCLC, including baicalein, wogonin, and oroxylin A [21]. However, to date, 126 small-molecule compounds have been isolated from HMM, and the functions of most of them are unknown [19]. Therefore, the components of HMM that can treat NSCLC warrant further investigation.

This study is devoted to investigate novel traditional and western drugs that could be combined to treat NSCLC. Firstly, we identified the 14 prognostic genes associated with ferroptosis in NSCLC patients. Then, single-cell sequencing results indicated the risk genes were mainly derived from lung tumor cells. Moreover, the drug sensitivity analysis showed 106 drugs correlating with ferroptosis-related genes. The traditional Chinese drugs of HMM were also identified in TCMSP database. Finally, the effects of concomitant drugs was validated by assays.

## Methods

### Data collection

The GSE31210 was downloaded from the GEO database as the test set, where contains 20 cases of normal samples and 226 cases of tumor samples were involved. The mRNA sequencing data on 1089 patients and the clinical information on corresponding samples were downloaded from the TCGA (LUAD and LUSC) database as the training set (involving 108 cases of normal samples and 1041 cases of tumor samples).

### DEG identification

Rank sum tests were performed on the training set by R V4.1.0. 587 ferroptosis-related genes were screened from previous literature reports. he data type used for DEG identification was the log2 transformed new FPKM and TPM value, namely, log2(FPKM + 1) and log2(TPM + 1). The data were normalized by using the limma package before DEG identification. Also, the new screening criteria were log FC ≥ 1 and adjusted *P* < 0.05. The intersection of the screened differential genes and iron death-related genes was taken, and the obtained results could be used for subsequent Univariate COX analysis and LASSO screening.

### Gene enrichment analysis

GSEA is a computational method for calculating and comparing the consistent differences present in two biological states. The differential analysis of the enrichment of intersecting genes was performed on different signaling pathways obtained from differential analysis by using the R package clusterprofiler. The gene enrichment in the corresponding pathway was significant (*P* < 0.05).

### Development and validation of prognostic risk models

The relationship between genes and overall survival (OS) was analyzed by using LASSO COX regression analysis. The software package was glmnet. A prognostic risk prediction model for NSCLC was established according to the LASSO risk score calculation formula. After the acquisition of the median risk score, NSCLC patients were divided into the high-risk and low-risk groups. The plotted KM curves were used for comparing OS between the two groups. The optimal R package for Survival analysis was the package SURVIVAL. The ROC curve analysis was adopted to assess the reliability of the prognostic model, and the evaluated standard for ROC curve analysis results (*P* < 0.05).

### Single-cell sequencing data analysis

The downstream analysis of the downloaded scRNA-seq data (GSE131907) was performed by using the Seurat R package (version 3.0.2). The number of cells with genes present in less than 3 or when the number of genes in a single cell is less than 200 were filtered out, with a limit of 20% for the percentage of mitochondria. The data processing needed to be normalized by the LogNormalize method. Subsequently, the marker genes were clustered by using the "FindAllMarkers" function with the filter condition set to (FC) ≥ 1. The minimum cell ratio in either of the two populations was 0.25. In addition, the expression pattern of each marker gene in the cluster was visualized. The expression pattern of genes in clusters was also visualized by applying Seagate's "DotPlot" function. Meanwhile, the SingleR package (version 1.0.0) was used for marker-based cell-type annotation.

### Protein level validation of central genes

HPA database, designed to create human proteome-wide maps by integrated OMC technology. To validate the protein expression levels of 14 genes, the protein expression of target genes from the HPA database was screened in different tissues of NSCLC patients.

### Drug sensitivity screening of therapeutic targets

The CellMiner database, a drug sensitivity database based on the NCI-60 cell line, was built by the National Cancer Institute (NCI). The RNA-seq and NIC-60 drug z-score values were downloaded from the CellMiner database (https://discover.nci.nih.gov/cellminer/home.do). Z-score values were positively correlated with drug sensitivity. FDA-approved drugs were obtained by screening, and Pearson correlation analysis was performed on the drug z-score values and characteristic gene expression. The threshold values were Pearson's correlation coefficient (PCC)|> 0.3 and *P* < 0.05.

### The collection of active ingredients of HMM

The Traditional Chinese Medicine Systems Pharmacology database (TCMSP) was investigated to acquire the ingredients of HMM (https://old.tcmsp-e.com/tcmsp.php). This database includes a set of ingredients, targeted genes, and pharmacokinetic properties of natural compounds. To get the active ingredients of HMM in the database, the screening criteria are oral bioavailability (OB) ≥ 30% and drug-likeness (DL) ≥ 0.18. 20 active ingredients and 206 targeted genes of HMM were collected.

### Molecular docking

Autodock vina (v1.2.0) was used to evaluate the molecular docking. The PDB files of proteins were downloaded from RSCB PDB (https://www.rcsb.org/). The mol2 file of small molecules were obtained from TCMSP and transformed into PDB files through Open Babel (v3.1.1). Then, the structure was introduced to Autodock vina for removing water, hydrogenation, charge and rotational bond number calculation and the lowest energy posture is selected for research. Pymol (v2.5.3) was performed to get the 3D-2D model of high-quality small molecules and proteins.

### Cell lines and cell culture

Lung cancer cell line A549 was provided by American type culture collection (ATCC). The A549 cells were cultured in McCoy’s 5A medium, supplemented with 10% bovine serum albumin (FBS), 100 μg/ml penicillin and 100 μg/ml streptomycin at 37 °C in 5% CO2.

### CCK8 assay to detect cell viability

CCK8 assay was used for cell viability identification. 5 × 10^3^ cells were inoculated into 96-well culture plates, and left adhering overnight. And then the cells were cultured in fresh medium containing different concentrations of CEP-9722 (MCE, HY-105303), RAF-265 (MCE, HY-10248), Gefitinib (MCE, HY-50895), BMS-599626 (MCE, HY-10251), and IMG (TargetMOI, CAS: 136087–29-1, Lot, 149,155) dissolved in DMSO (final concentration, 0.1%). After 48 h of incubation, CCK-8 was added and the absorbance at 450 nm was measured with an EnSpire® Multi-plate Reader (Perkin Elmer, USA). Each group of experiments was repeated 3 times.

### Colony formation assay

The assay was conducted to analyze the effect of CEP-9722 on colony formation. Single cells were cultured in six-well plates with a cell count of 1 × 10^3^ per well for 24 h. After 24 h, the medium was removed and different concentrations of CEP-9722 and IMG were added, and the cells were continued to be cultured until the colonies were clearly visible. Finally, the cells were stained with 0.1% crystal violet for counting.

### Iron assay

The iron assay was conducted through the iron assay kit (ab83366, Abcam). The A549 cells were cultured in a 10 cm^2^ plate with a cell count of 5 × 10^6^ for 24 h. After 24 h, the cells were treated with IMG, CEP-9722, erastin, RSL3, or DMSO for 12 h. Then, the cells were collected in the 5 × volumes of iron assay buffer on ice according to the manufacturer’s instructions. To remove insoluble material, the cells were centrifuged with 13000 g, 10 min at 4℃. The supernatant was added to the iron reducer and incubated at room temperature for 30 min. Next, 100 ul of the iron probe was added and incubated at room temperature for 1 h. The absorbance at 593 nm was detected using a colorimetric microplate reader.

### Statistical analysis

Statistical analyses were performed with KM curves to compare the difference in survival between two risk groups. The predictive power of the model was determined by ROC curves analysis. Univariate COX regression analysis was adopted to assess the prognostic value of risk scores, with hazard ratios (HR) and 95% confidence intervals (CI) set for each variable. All parameters were default and there was a significant difference (*P* < 0.05).

## Results

### Ferroptosis-related differential genes in NSCLC

**Fig. 1:**
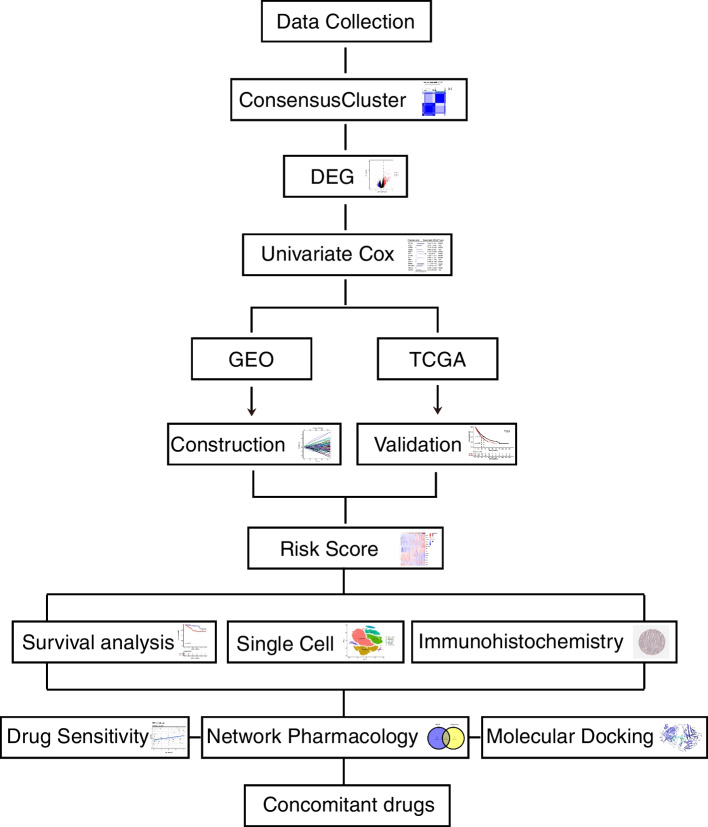
Flow chart of the study

**Fig. 2:**
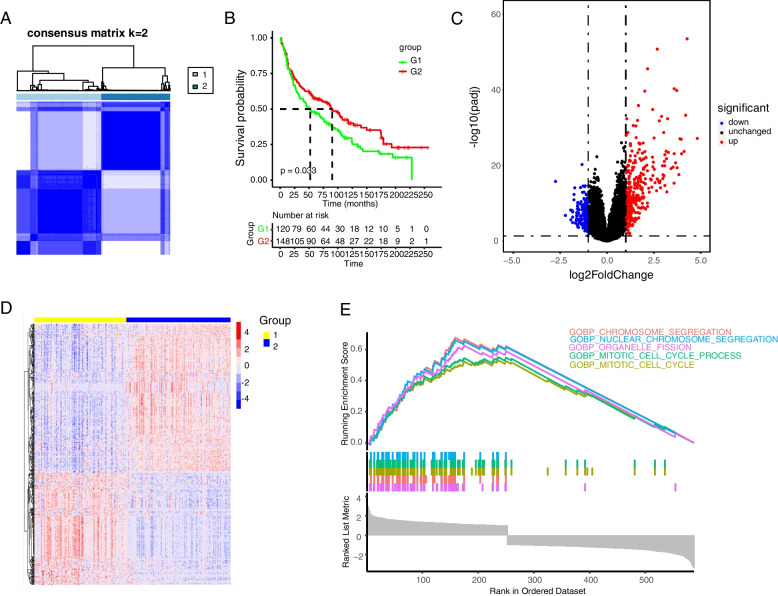
Differentially expressed gene analysis of NSCLC patients based on ferroptosis-associated clusters. **A** The consensus matrix of all samples when k = 2. **B** Kaplan–Meier curves of patients in 2 ferroptosis-associated clusters for overall survival. **C** Volcano plot and **D** heatmap of ferroptosis-associated DEGs between cluster 1 and cluster 2. **E** GSEA analysis of DEGs between 2 cluster groups

The flow chart is shown in Fig. 1. Based on the expression values of 259 ferroptosis-related genes, the data from GSE31210 database involving 226 samples were divided into k groups (k = 2–9) by cluster analysis through the Consensus Cluster Plus R package. The most significant effect was observed when k = 2 (Fig. 2A). The results of the PCA plots further demonstrated the accuracy of the cluster analysis (Supplementary Fig. 1A). Many studies have suggested that ferroptosis can inhibit cancer progression and improve the survival time of patients (13,14). The survival analysis result revealed that the overall survival time in group 2 was longer than that in group 1 (Fig. 2B), indicating that cluster group 2 has higher levels of ferroptosis. Furthermore, we analyzed the differentially expressed genes in tumor samples based on the ferroptosis-related cluster groups and got 252 up-regulated and 335 down-regulated genes (Fig. 2C, D). GSEA analysis indicates that the differentially expressed genes main enrich in chromosome segregation, nuclear chromosome segregation, organelle fission, and cell cycle (Fig. 2E and Supplementary Fig. 1B).

### Identification of prognostic models for genes associated with ferroptosis

**Fig. 3:**
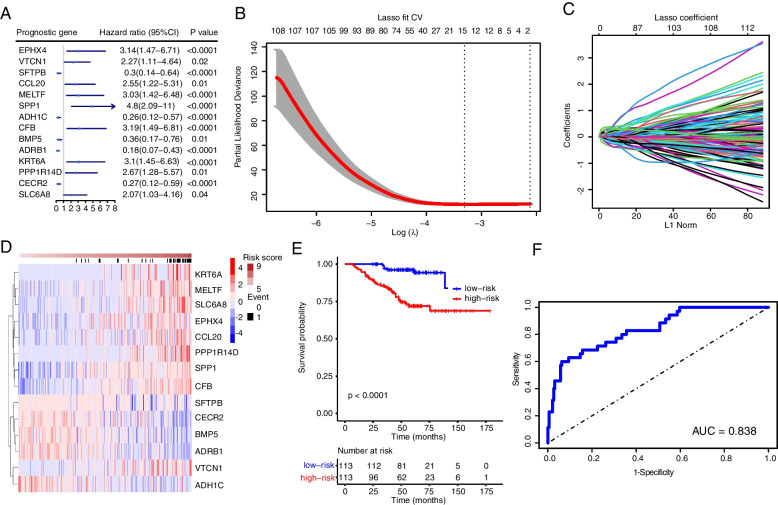
Construction of a risk-prediction model of the training set and analyses of model performance. **A** Univariate Cox regression analysis of risk score genes correlated with ferroptosis. **B** LASSO regression coefficient profile of the 369 intersection genes. **C** LASSO deviance profile of the 369 intersection genes. **D** Risk score in the GEO test set, patient survival, and expression of 14 DEGs in the test set. **E** Kaplan–Meier curves of patients in 2 ferroptosis-associated clusters for overall survival. **F** ROC curve of the risk score model

Firstly, the gene expression matrix of 226 patients was obtained by filtering out the samples from the GSE31210 database with no survival time or survival time less than 30 days. Then, the prognostic value of ferroptosis-related differentially expressed genes was assessed by univariate COX regression analysis. By setting the screening criterion *P* < 0.05, 369 genes associated with NSCLC prognosis were obtained (Supplementary Table S1). Further, the LASSO regression model was further used for the obtained expression values of prognostic genes to reduce the expression matrix of 369 prognostic genes. Finally, 14 gene markers were screened by the minimum λ value (λ), with the acquisition of the coefficients and hazard ratios of corresponding genes (Fig. 3A-C). The results are shown in Table 1. It was concluded that for genes with HR < 1, their corresponding genes are protective, with a better prognosis. On the contrary, genes with HR > 1 are risk genes, namely, the ones that may negatively affect prognosis. In addition, the risk scores of these genes in NSCLC patients can serve as markers for predicting prognosis.

**Table 1:** The risk score genes of NSCLC

gene	value
EPHX4	0.010178
VTCN1	0.126914
SFTPB	0.028276
CCL20	0.016218
MELTF	0.001586
SPP1	0.06785
ADH1C	-0.1536
CFB	0.289007
BMP5	-0.00874
ADRB1	-0.00709
KRT6A	0.021132
PPP1R14D	0.156499
CECR2	-0.04418
SLC6A8	0.196748

The risk score = 0.010178*EPHX4 + 0.126914*VTCN1 + 0.028276*SFTPB + 0.016218*CCL20 + 0.001586*MELTF + 0.06785*SPP1-0.1536*ADH1C + CFB*0.289007–0.00874*BMP5-0.00709*ADRB1 + 0.021132*KRT6A + 0.156499*PPP1R14D-0.04418*CECR2 + 0.196748*SLC6A8.

The heatmap analysis results showed the expression values of genes with prognosis-related risk scores were about survival (Fig. 3D). Based on the median expression values, the data inside the training set were divided into a high-risk group (*n* = 113) and a low-risk group (*n* = 113), but the graph displayed that the samples of samples low-risk group have a preferable prognosis (Fig. 3E and Supplementary Fig. 2C). The model was found to have good predictive power by ROC curve and PCA analyses. (Fig. 3F and Supplementary Fig. 2A, B).

### Validation of risk score in prognostic models

**Fig. 4:**
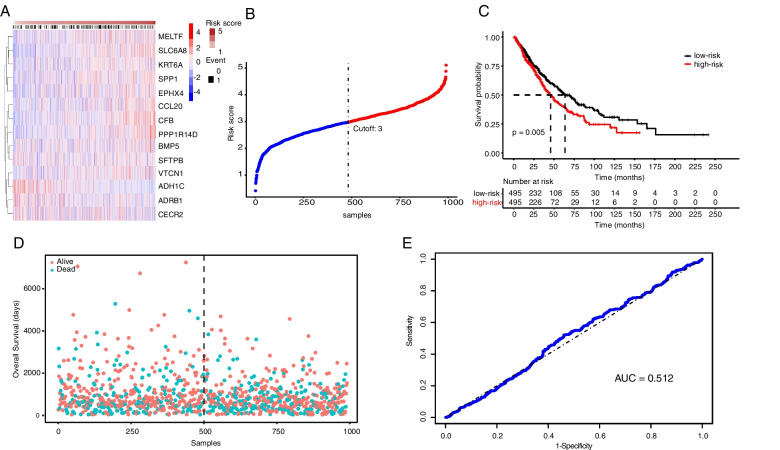
Validation of a risk-prediction model in test dataset. **A** Risk score in the TCGA test set, patient survival, and expression of 14 DEGs in the test set. **B** The risk scores of NSCLC in TCGA database. **C** Kaplan–Meier curves of patients in 2 ferroptosis-associated clusters for overall survival. **D** The distribution of survival status and risk scores in NSCLC patients. **E** ROC curve of the risk score model

The risk scores of risk genes already obtained in TCGA were used to evaluate the accuracy of the model (Fig. 4A, B). In the different datasets, the risk scores were calculated, and the patients were divided into high and low expression groups according to the median gene expression. And in the TCGA test set, the low expression group was found to have a better prognosis than the high expression group (Fig. 4C). Also, the ROC curve analysis indicated the risk model has a certain predictive value (Fig. 4D-E). In summary, it was believed that our prediction model can improve the ability to predict this disease in clinical applications. To further evaluate the accuracy of the risk score model, the GEO data of GSE30219 was used to validate this model. The results were similar to the TCGA data (Supplementary Fig. 3). Besides, the patients in the data of GSE31210 used to construct the predictive model were mainly derived from Japan. Therefore our risk score model is mainly used to predict the survival status of the East Asian race.

### Biological function of 14 prognostic genes revealed by single-cell sequencing

**Fig. 5:**
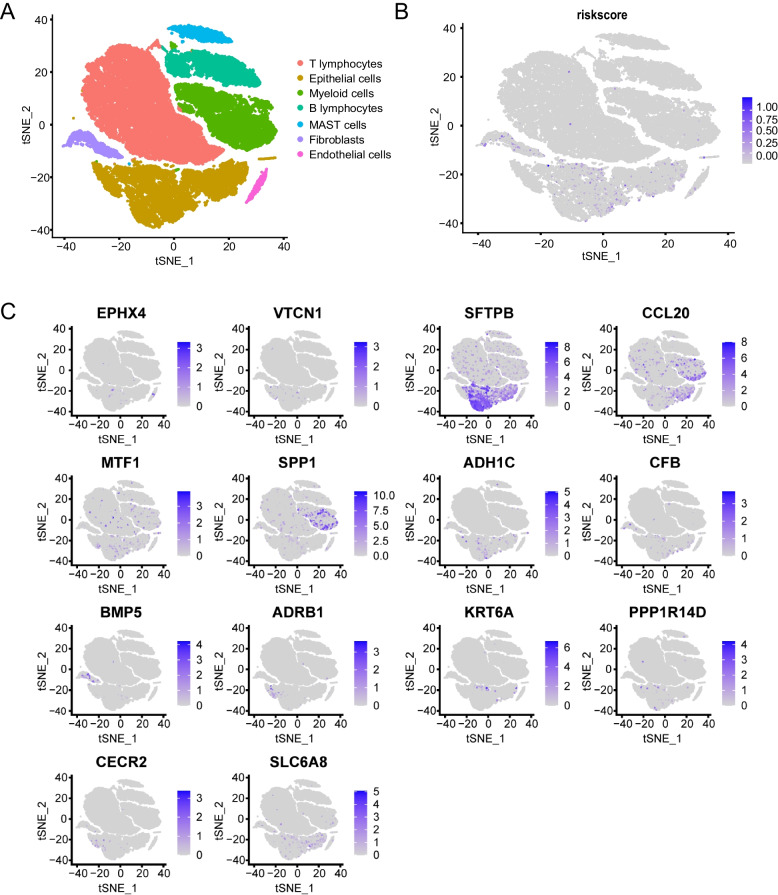
The results of risk score and prognostic genes in NSCLC patients by using scRNA-seq. **A** The cells were categorized into 7 clusters based on different cell markers. **B** Scatter plots show the risk score expression distribution in different cell clusters. **C** The distributions of signature genes in different cell populations in NSCLC

The characteristics and risk scores of 14 prognostic genes were further used for single-cell sequencing analysis. First, it was found that 14 prognostic genes had high expression in epithelial cells (cell fractionation type) by fractionating tumor cells with already annotated cell types [22] (Fig. 5A and Supplementary Fig. 4 and Table S2). Then, we used the prediction model to calculate the risk score values of single cell data. The result showed that the risk score values mainly enrich in the epithelial cells (lung cancer cells). In addition, some risk values are also enriched in fibroblasts (Fig. 5B, C). Compared with other cell types, the genes SPP1 and BMP5 were down-regulated in the epithelial cells, while the other risk score genes were up-regulated.

### Validation of pivotal genes (prognostic genes) at the proteomic level

**Fig. 6:**
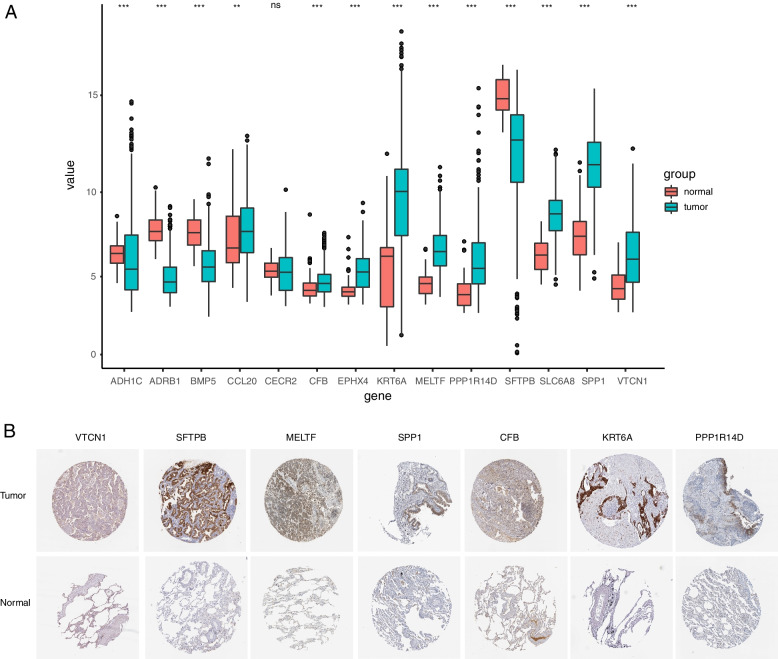
Validation of prognostic genes at the proteomic level. **A** The boxplot showed the expression of 14 hub genes between tumor and normal tissues in TCGA database. **B** The protein levels of hub genes were presented by immunohistochemical staining analysis from Human Protein Atlas database

The expression of 14 genes was significantly different in tumor and normal tissues (Fig. 6A). The prognostic models for iron death-related genes have been obtained by LASSO and they were validated in the test group. The previous analysis results were mainly based on the transcriptomic data. In order to further investigate the expression levels of relevant prognostic genes in the proteome, the HPA database was used for screening of important genes and 7 proteins were found to have a higher expression in lung cancer tissue (Fig. 6B and Supplementary Fig. 5A-D).

### Drug sensitivity analysis based on prognostic models

**Fig. 7:**
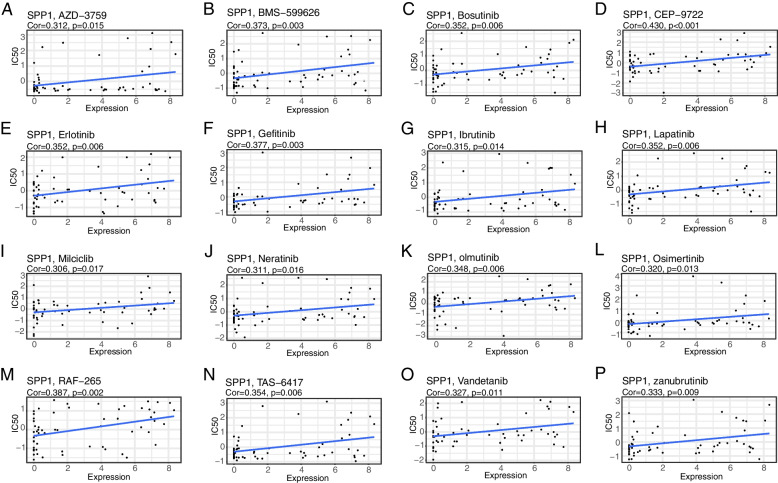
Drug sensitivity analysis of prognostic factors. **A**-**P** The correlation scatter plot between the expression level of prognostic predictors and the drug IC50 value in CellMiner database was selected, and 16 groups of relationships were detected

CellMiner, a web-based genomic and pharmacology database, provides a way to analyze drugs and genomics. The data on 709 drugs (Z-score normalized) approved by clinical trials and/or the FDA were screened and retained. By comparison with the gene set in the HL60 data, 14 genes were retained for analysis. In the second step, 630 results were obtained by correlating the expression levels of the screened 14 genes with the GI50 data on the drugs in CellMiner database (Fig. 7A-P and Supplementary Fig. 6A-P, Supplementary Table S3). It was found that SPP1 genes were significantly and positively correlated with the expression of the drug CEP-9722, indicating that with SPP1 expression increasing, the semi-inhibitory concentration of the drug CEP-9722 decreases, which can improve the therapeutic effect (Fig. 7D and Supplementary Fig. 6D).

### Identify the effective ingredients of HMM by network pharmacologyand molecular docking

**Fig. 8:**
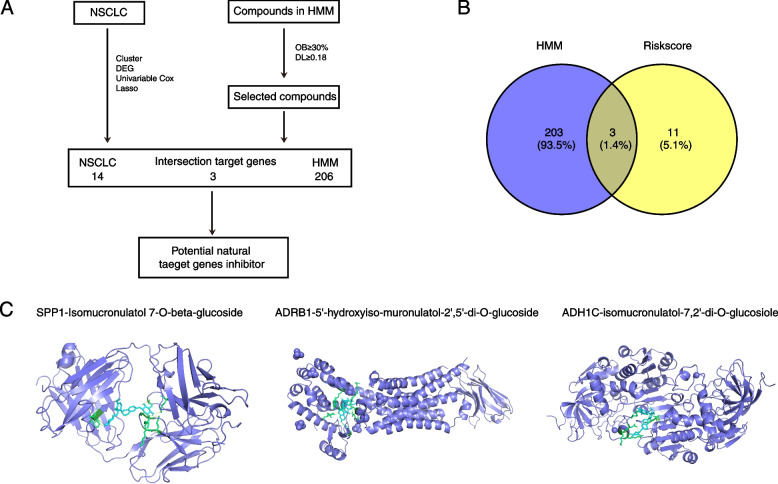
Network pharmacology and molecular docking of active ingredients of HMM and ferroptosis-related signature genes. **A** The flow of network pharmacology and molecular docking. **B** The overlap genes between 206 targeted genes of active ingredients in HMM and 14 ferroptosis-related biomarkers. **C** The 3D structure model of small molecule and protein

To investigate the traditional Chinese drugs available in NSCLC, we collected 20 active ingredients of HMM from the TCMSP database (Fig. 8A and Table 2). The screening criteria are OB ≥ 30% and DL ≥ 0.18. Firstly, we got 206 targeted genes of these 20 active ingredients (Supplementary Table S4). The overlap of the targeted genes and 14 risk genes(Fig. 8B), SPP1, ADRB1, and ADH1C, were evaluated with the 20 active ingredients through molecular docking, and the results showed that isomucronulatol 7-O-beta-glucoside and 5’-hydroxyiso-muronulatol-2’,5’-di-O-glucoside have the highest vina scores with these genes (Fig. 8C*,* Supplementary Fig. 7A-C, and Supplementary Table S5).

**Table 2:** Characteristics of active ingredients in HMM

No	Molecule ID	Molecule name	Molecular weight	OB (%)	DL
1	MOL000211	Mairin	456.78	55.38	0.78
2	MOL000239	Jaranol	314.31	50.83	0.29
3	MOL000296	hederagenin	414.79	36.91	0.75
4	MOL000033	(3S,8S,9S,10R,13R,14S,17R)-10,13-dimethyl-17-[(2R,5S)-5-propan-2-yloctan-2-yl]-2,3,4,7,8,9,11,12,14,15,16,17-dodecahydro-1H-cyclopenta[a]phenanthren-3-ol	428.82	36.23	0.78
5	MOL000354	isorhamnetin	316.28	49.6	0.31
6	MOL000371	3,9-di-O-methylnissolin	314.36	53.74	0.48
7	MOL000374	5'-hydroxyiso-muronulatol-2',5'-di-O-glucoside	642.67	41.72	0.69
8	MOL000378	7-O-methylisomucronulatol	316.38	74.69	0.3
9	MOL000379	9,10-dimethoxypterocarpan-3-O-β-D-glucoside	462.49	36.74	0.92
10	MOL000380	(6aR,11aR)-9,10-dimethoxy-6a,11a-dihydro-6H-benzofurano[3,2-c]chromen-3-ol	300.33	64.26	0.42
11	MOL000387	Bifendate	418.38	31.1	0.67
12	MOL000392	formononetin	268.28	69.67	0.21
13	MOL000398	isoflavanone	316.33	109.99	0.3
14	MOL000417	Calycosin	284.28	47.75	0.24
15	MOL000422	kaempferol	286.25	41.88	0.24
16	MOL000433	FA	441.45	68.96	0.71
17	MOL000438	(3R)-3-(2-hydroxy-3,4-dimethoxyphenyl)chroman-7-ol	302.35	67.67	0.26
18	MOL000439	isomucronulatol-7,2'-di-O-glucosiole	626.67	49.28	0.62
19	MOL000442	1,7-Dihydroxy-3,9-dimethoxy pterocarpene	314.31	39.05	0.48
20	MOL000098	quercetin	302.25	46.43	0.28

### The suppression of the proliferation of human lung cancer cells by CEP-9722 and IMG

**Fig. 9:**
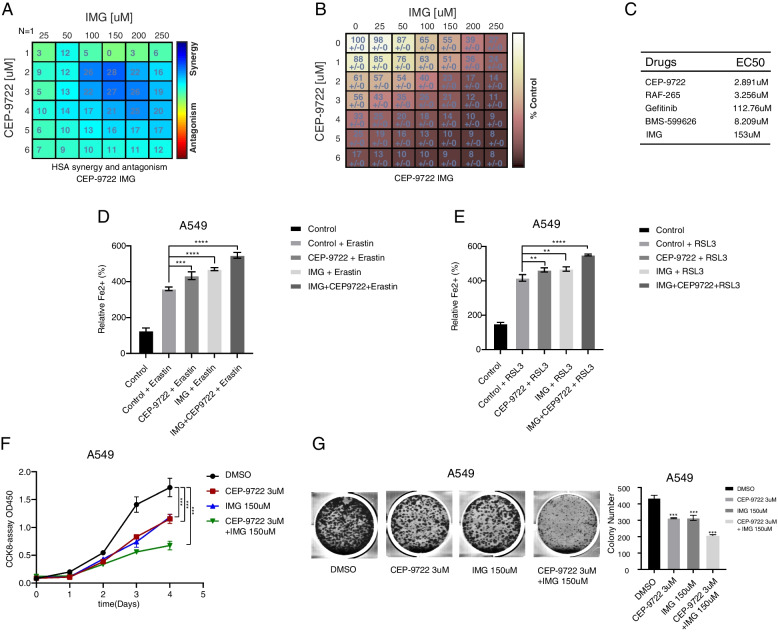
The effect of cell proliferation in NSCLC cell lines by using CEP-9722 and IMG. **A** The HAS synergy and antagonism of CEP-9722 and IMG in A549 cell. **B** The cell percentages of A549 treated with CEP-9722 and IMG. **C** The IC50 values of CEP-9722, RAF-265, Gefitinib, BMS-599626, and IMG for cell viability inhibition in lung cancer cell line A549 were determined. **D**, **E** Control group represents DMSO-treated cells. **F** The CCK8 assay of A549 cell treated with different drugs. **G** A549 cells were cultured with CEP-9722 and/or IMG. Analyzing the induced colony formation and calculate the colony formation. Data shown represent mean ± SD from three independent experiments. **p < 0.01; ***p < 0.001; ****p < 0.0001

First, the effect of CEP-9722, RAF-265, Gefitinib, BMS-599626, and IMG on the proliferation of cell lines A549 was examined by CCK8 assay (Supplementary Fig. 8A-E). Also, the IC50 values of the drugs in A549 were calculated (Fig. 9C). The results revealed that A549 was more sensitive to drug CEP-9722 treatment, compared to other agents. Due to the drug CEP-9722 targeting SPP1, the traditional drug IMG was selected to target the same gene, and the combination of CEP-9722 and IMG was further investigated (Fig. 9A, B). Then, we invested the concentrations of Fe^2+^ in erastin- and RSL3-treated A549 cells by using CEP-9722 and/or IMG. The results showed that the combination of CEP-9722 and IMG increased the ferroptosis of A549 cells (Fig. 9D, E). Moreover, we treated A549 cell with 3uM and 150uM effective concentrations of CEP-9722 and IMG for further mechanistic studies. As Fig. 9F-G shows, the combination of CEP-9722 and IMG has a stronger inhibition effect than the drugs used alone. These results suggested that CEP-9722 and IMG has a significant synergy effect for inhibiting the growth of lung cancer.

## Discussion

NSCLC is a common subtype of lung cancer. In current clinical treatment, advanced patients with poor prognosis and short survival time, and lack of effective targeted therapy are the most serious challenges [23, 24]. As a type of regulated death, the role of ferroptosis should not be ignored in regulating cell development. It has been already reported that ferroptosis-related genes have been investigated as disease prediction models in multiple cancers [25]. However, the therapy of combining traditional and western medicine by ferroptosis in NSCLC patients have not yet been fully elucidated. Therefore, based on LASSO regression, it is necessary to establish a ferroptosis-related risk score model to evaluate the prognosis of NSCLC patients. Then, the expression of risk scores in various subtypes of NSCLC was obtained by analyzing the single-cell sequencing data. Finally, the drug sensitivity analysis of potential targets through the CellMiner database showed that CEP9722 had the most significant tumor-suppressive effect. Meanwhile, network pharmacology and molecular docking analysis were performed to investigate the pontential active ingredients of HMM targeting ferroptosis-related genes, and the combination of traditional and western drugs were used to examine the effects for treating NSCLC. In conclusion, this study provides a potent strategy of concomitant drugs with traditional and western medicine for treating NSCLC.

Ferroptosis has long been considered as an iron-dependent death caused by membrane damage mediated by an imbalance of redox reactions in vivo [26]. It has become a new therapeutic direction that tumor cell growth is regulated by developing new drugs to trigger the ferroptosis pathway [27]. Ferroptosis-related genes have been reported as biomarkers in many studies. For example, Ren and Han et al. have investigated ferroptosis genes as biomarkers in both lung adenocarcinoma and NSCLC [25, 28]. However, in their study, the drugs targeting predicted genes and single-cell data analysis were not elucidated. In NSCLC patients, 259 ferroptosis-related genes were clustered and the grouping effect was found to be the best when k = 2. Through differential gene expression analysis of patients, 587 differential genes were obtained. Then, by using LASSO linear regression model to perform Univariate COX and LASSO analyses on the ferroptosis-associated genes, a prognostic model of 14 ferroptosis-related genes was obtained. And 14 genes were found to be significantly associated with clinical prognosis. SPP1 can act as a hub gene for methylation and enhance colorectal cancer (CRC) metastasis through the mesenchymal transition in CRC cell lines [29]. Additionally, silencing SPP1 expression was found to inhibit the growth, migration, and cell cycle of tumors. Furthermore, SPP1-encoded proteins, including fibronectin 1 and osteopontin (OPN) [30], are involved in processes such as wound healing and angiogenesis and they are closely associated with tumor prognosis [29]. Finally, the predictive function of the model was comprehensively evaluated by ROC curve, PCA analysis. In summary, we comprehensively evaluate and verify the performance of the model through a variety of methods.

The rise of single-cell sequencing technologies based on tumor cell heterogeneity is a technological advance, which provides a powerful tool for further revealing molecular mechanisms. Advances in single-cell sequencing technologies have provided the possibility of identifying novel or rare cell types, analyzing single-cell trajectory construction, and comparing healthy and disease-related tissue at single-cell resolution [31]. Meanwhile, single-cell sequencing has great development potential for promoting the diagnosis, targeted therapy, and prognosis prediction of various tumors [32]. Cell subtypes have been grouped and validated by single-cell sequencing based on prognostic models in tumors. However, previous ferroptosis-related prediction models have not been validated by single-cell sequencing. Therefore, cell clustering and trajectory analyses were performed on 14 prognostic genes based on single-cell sequencing data from NSCLC. First, according to the single-cell sequencing data on NSCLC, the cells were divided into seven categories: T lymphocytes, epithelial cells, myeloid cells, B lymphocytes, MAST cells, fibroblasts, and endothelial cells. Among them, the epithelial cells were found to have the highest risk score. The feasibility of 14 ferroptosis-related genes for prognostic models was further verified.

Through the CellMiner database, a chemotherapeutic drug sensitivity analysis was conducted, and the results indicates that these signature genes were positively correlated with 98 chemotherapeutic drugs. This suggested that our model can be used as a postoperative adjuvant chemotherapy model for prediction. In order to further improve the clinical predictive value, the most relevant drug CEP-9722 was selected for verification at the cellular level, confirming that the drug CEP-9722 has a significant inhibitory effect on NSCLC. The current treatment for NSCLC patients is mainly immunotherapy based on antibodies against PD-1 or PD-L1 [33]. Although the current treatment methods for NSCLC patients are greatly improved, patients with advanced cancer will still develop resistance to the tumor treatment. Thus, it is important to find a new therapy. Our prediction model was used to perform the screening for CEP-9722, a prodrug of CEP-8983, which is a potent inhibitor of PARP-1 and PARP-2 [17]. Both PARP-1 and PARP-2 are the major pathways for DNA repair in tumor cells [17]. CEP-9722 can further promote the effect of DNA-damaging chemotherapy by inhibiting two major pathways. Besides, CEP-9722 can enhance the sensitivity of chemotherapy-resistant tumor cells as monotherapy or combined with other drugs [34]. Moreover, the active ingredients of HMM were collected from the TCMSP database and their targeted genes were evaluated. Through overlapping these targeted genes with ferroptosis-related signature genes of NSCLC, we identified 3 genes targeted by active ingredients of HMM and correlated with ferroptosis and prognosis. Altogether, the findings in this study provide a potential strategy for concomitant drugs to treat NSCLC.

## Conclusions

Our study provided a novel strategy for concomitant drugs to treat NSCLC associated with ferroptosis biomarkers. The risk score genes were mainly contributed by the tumor cells. Among these genes, SPP1-sensitive drug CEP-9722 and IMG was validated to have a synergy effect in NSCLC cells. Therefore, the potential strategy of concomitant drugs is devoted to exploring clinical therapeutic effects for NSCLC.

## Supplementary Information

**Additional file 1: Figure S1.** PCA and GO results. Principal component analysisof patients in 2 ferroptosis-associated clusters.The representative results of the Gene Ontology analysis between 2 cluster groups.

**Additional file 2: Figure S2.** Test set results. The risk scores of NSCLC in GEO31210 database. The PCA analysis of patients with high- and low-risk score groups. The distribution of survival status and risk scores in NSCLC patients.

**Additional file 3: Figure S3.** Validation of a risk-prediction model in test dataset GSE30219. Risk score in the GSE30219 test set, patient survival, and expression of DEGs in the test set. The risk scores of NSCLC in TCGA database. Kaplan-Meier curves of patients in 2 ferroptosis-associated clusters for overall survival. The distribution of survival status and risk scores in NSCLC patients. ROC curve of the risk score model.

**Additional file 4: Figure S4.** The distributions of signature genes in different cell populations.

**Additional file 5: Figure S5.** HPA results. The protein levels of ADH1C, ADRB1, CECR2, and EPHX4 in HPA database.

**Additional file 6: Figure S6.** CellMiner results.the boxplot of the differences in drug sensitivity between the two clusters was shown.

**Additional file 7: Figure S7.** Molecular docking results. The amplification image of 3 prognostic genes with active ingredients. SPP1 and isomucronulatol 7-O-beta-glucosideADRB1 and 5’-hydroxyiso-muronulatol-2’,5’-di-O-glucosideADH1C and isomucronulatol 7-O-beta-glucoside.

**Additional file 8: Figure S8.** The CCK8 results of A549 cells withCEP-9722,RAF-265,Gefitinib,BMS-599626,IMG.

**Additional file 9: Table S1.** The COX analysis results of 369 genes associated with NSCLC.

**Additional file 10: Table S2.** The top 20 marker genes of the single cell data of NSCLC.

**Additional file 11: Table S3.** The correlation of 14 signature genes with drugs.

**Additional file 12: Table S4.** The 206 target genes of HMM active ingredients.

**Additional file 13: Table S5.** Molecular docking parameters and results of active ingredients in HMM binging with risk score genes.

**Additional file 14.** The IMG HNMR report from TargetMOI.

## Data Availability

The datasets used in this study are available from the TCGA database (https://xenabrowser.net/datapages/), GEO database (https://www.ncbi.nlm.nih.gov/), the HPA database (https://www.proteinatlas.org/), the CellMiner database (https://discover.nci.nih.gov/cellminer/home.do), the TCMSP databse (https://old.tcmsp-e.com/tcmsp.php), and the RCSB PDB database (https://www.rcsb.org/).
