# Supplementary figures and images for: The combination therapy of isomucronulatol 7-O-beta-glucoside (IMG) and CEP-9722 targeting ferroptosis-related biomarkers in non-small cell lung cancer (NSCLC)
Source: BMC Pulm Med. 2023 May 11;23:162. doi: 10.1186/s12890-023-02445-0 (PMC10173508; doi:10.1186/s12890-023-02445-0)

A

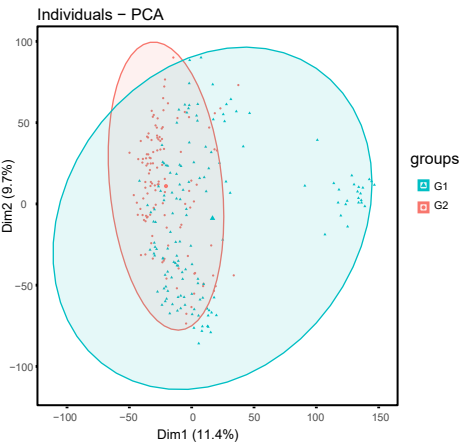

B

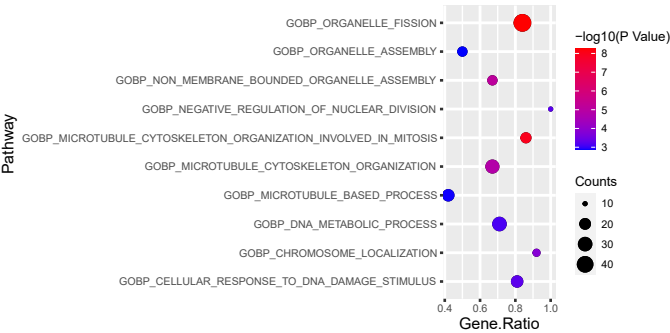

Supplement: Supplementary file 1 — Additional file 1: Figure S1. PCA and GO results. Principal component analysisof patients in 2 ferroptosis-associated clusters.The representative results of the Gene Ontology analysis between 2 cluster groups. [file 12890_2023_2445_MOESM1_ESM.pdf]

A

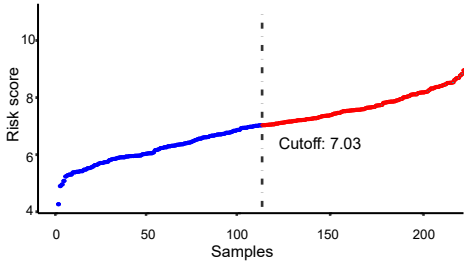

B

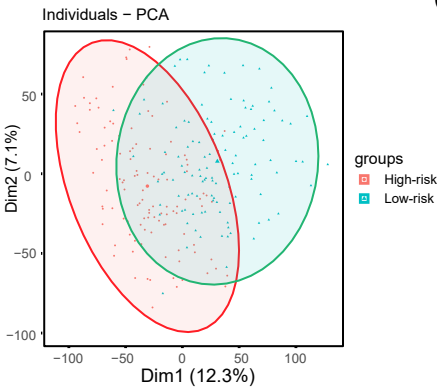

C

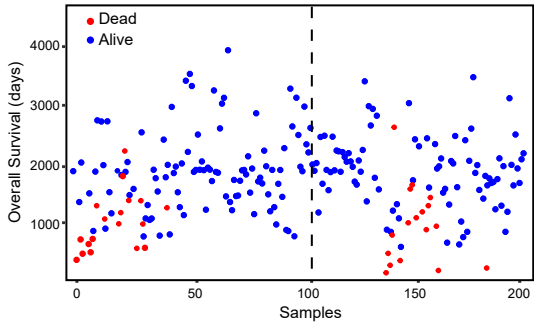

Supplement: Supplementary file 2 — Additional file 2: Figure S2. Test set results. The risk scores of NSCLC in GEO31210 database. The PCA analysis of patients with high- and low-risk score groups. The distribution of survival status and risk scores in NSCLC patients. [file 12890_2023_2445_MOESM2_ESM.pdf]

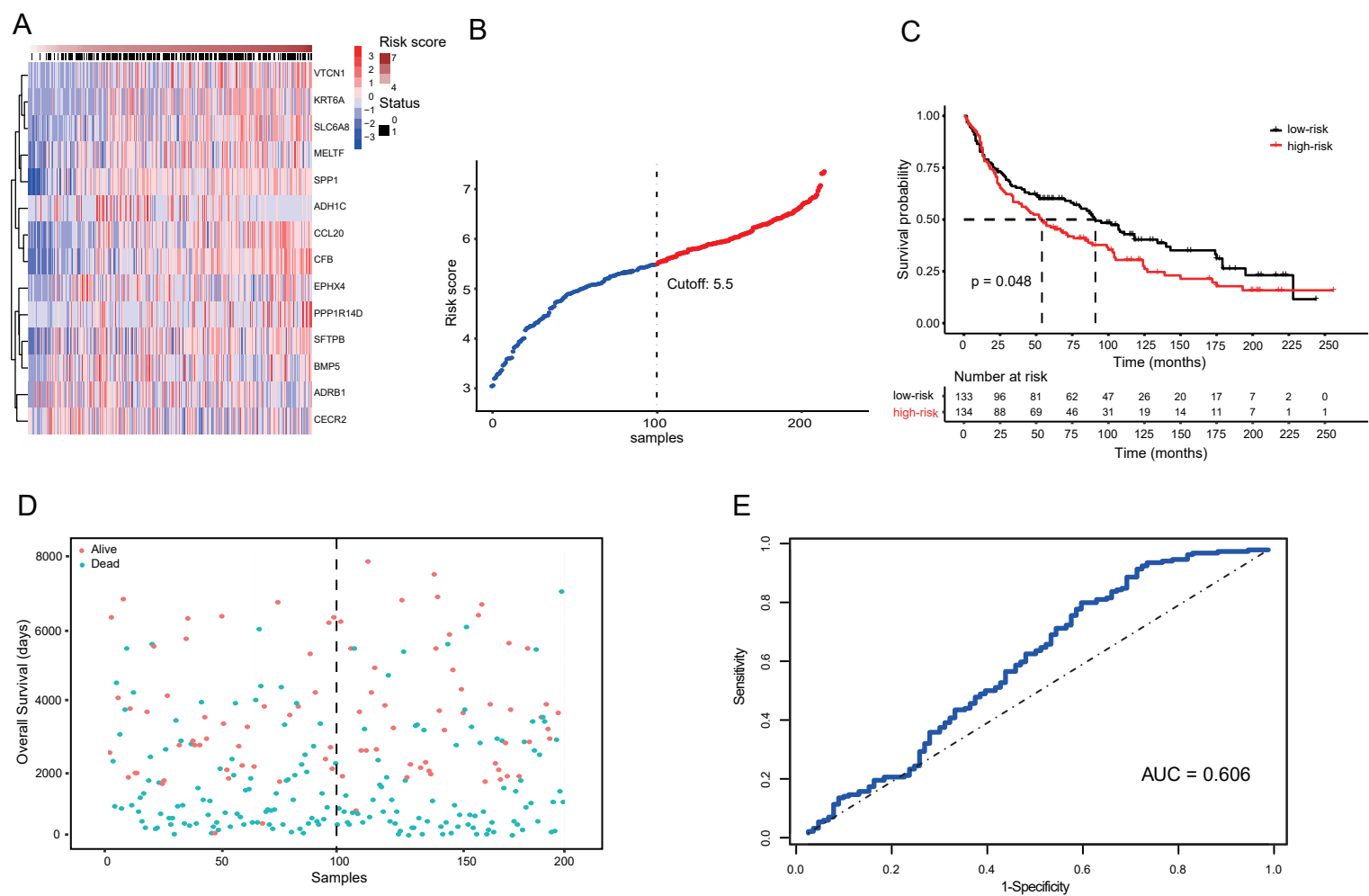

Supplement: Supplementary file 3 — Additional file 3: Figure S3. Validation of a risk-prediction model in test dataset GSE30219. Risk score in the GSE30219 test set, patient survival, and expression of DEGs in the test set. The risk scores of NSCLC in TCGA database. Kaplan-Meier curves of patients in 2 ferroptosis-associated clusters for overall survival. The distribution of survival status and risk scores in NSCLC patients. ROC curve of the risk score model. [file 12890_2023_2445_MOESM3_ESM.pdf]

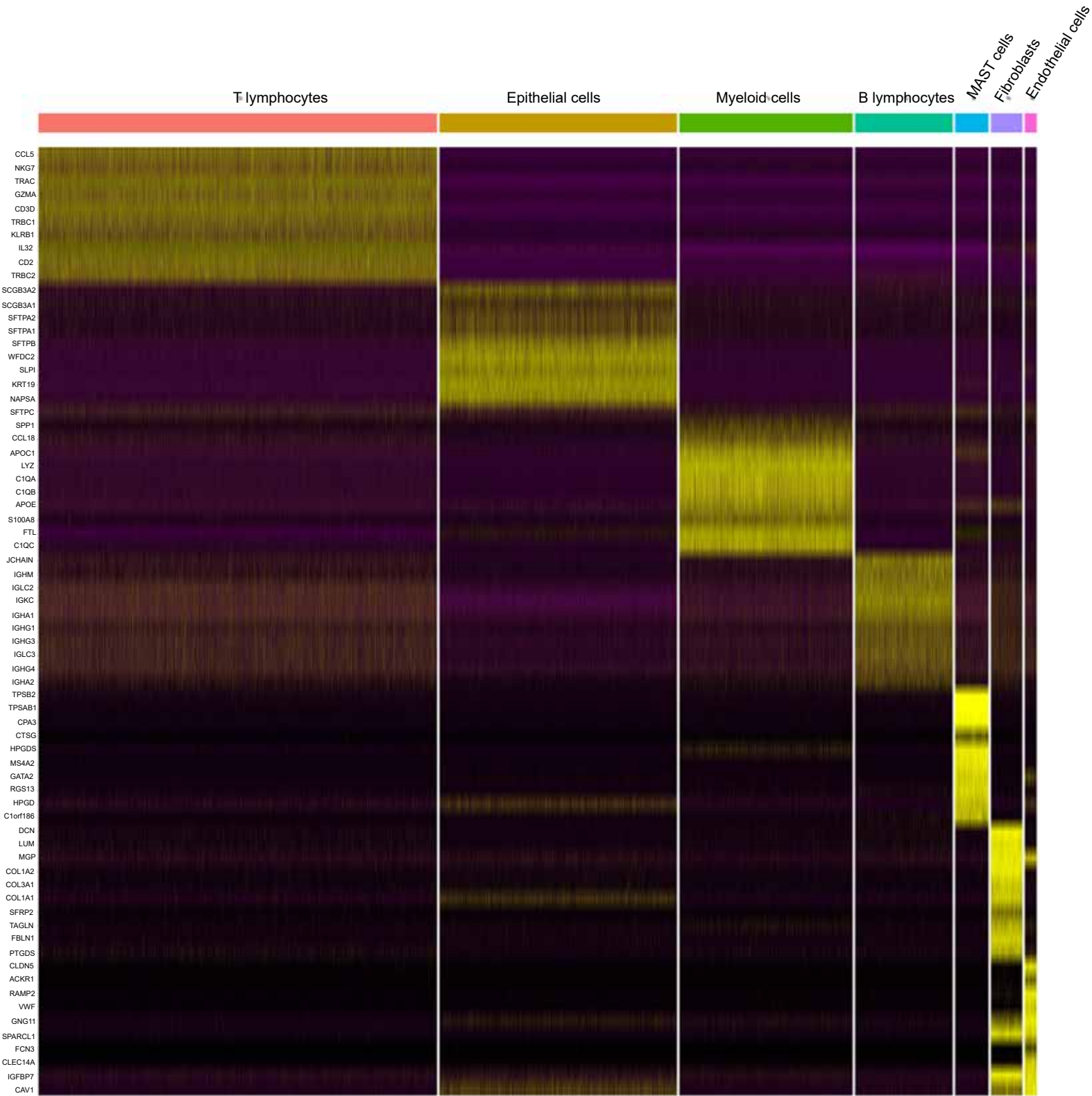

Supplement: Supplementary file 4 — Additional file 4: Figure S4. The distributions of signature genes in different cell populations. [file 12890_2023_2445_MOESM4_ESM.pdf]

**A**

ADH1C

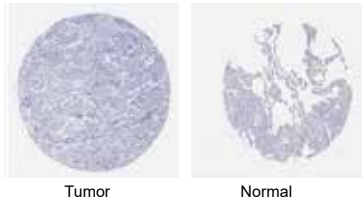

**B**

ADRB1

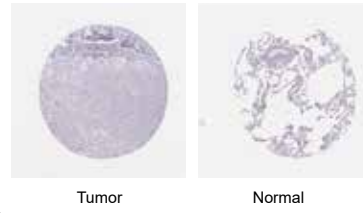

**C**

CECR2

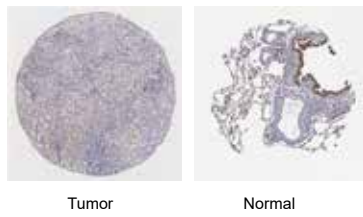

**D**

EPHX4

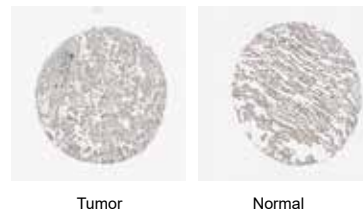

Supplement: Supplementary file 5 — Additional file 5: Figure S5. HPA results. The protein levels of ADH1C, ADRB1, CECR2, and EPHX4 in HPA database. [file 12890_2023_2445_MOESM5_ESM.pdf]

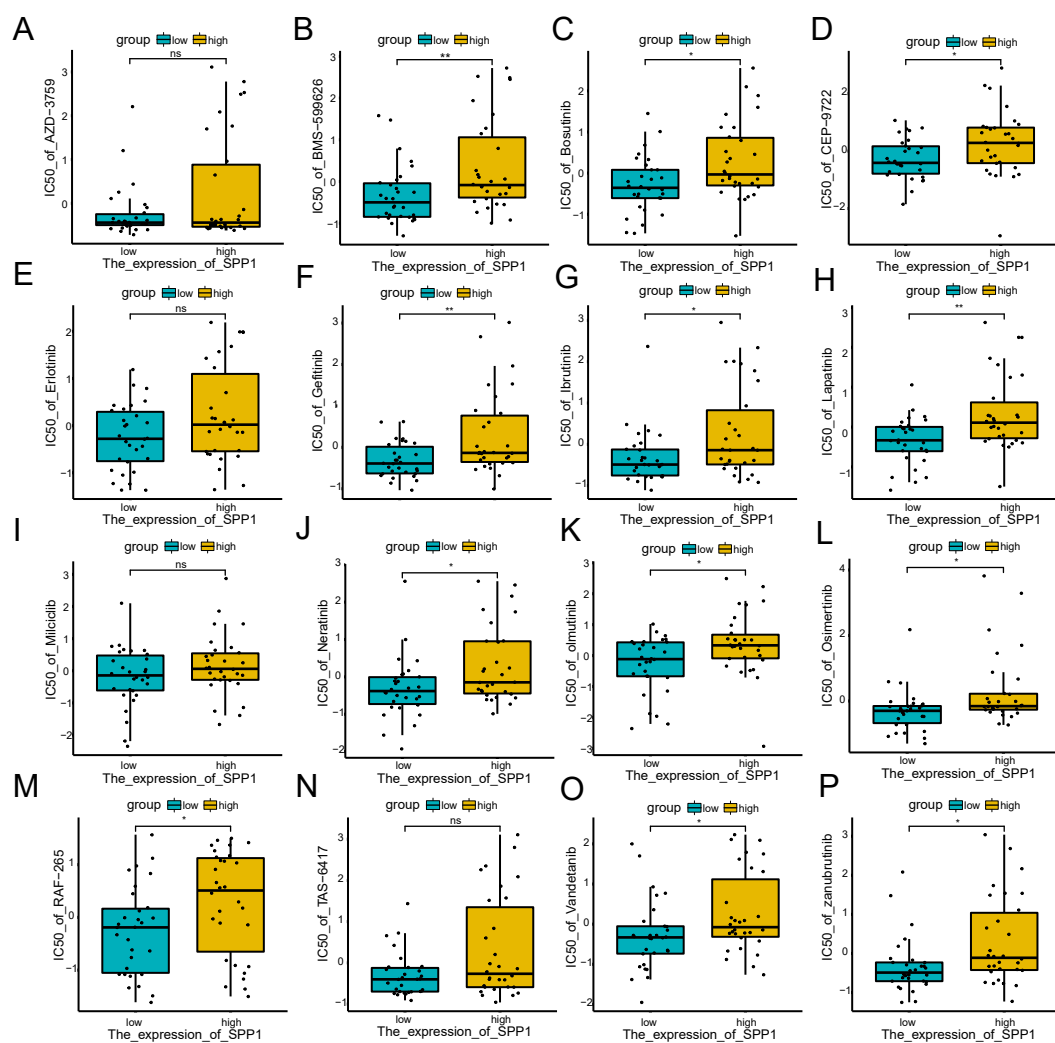

Supplement: Supplementary file 6 — Additional file 6: Figure S6. CellMiner results.the boxplot of the differences in drug sensitivity between the two clusters was shown. [file 12890_2023_2445_MOESM6_ESM.pdf]

A

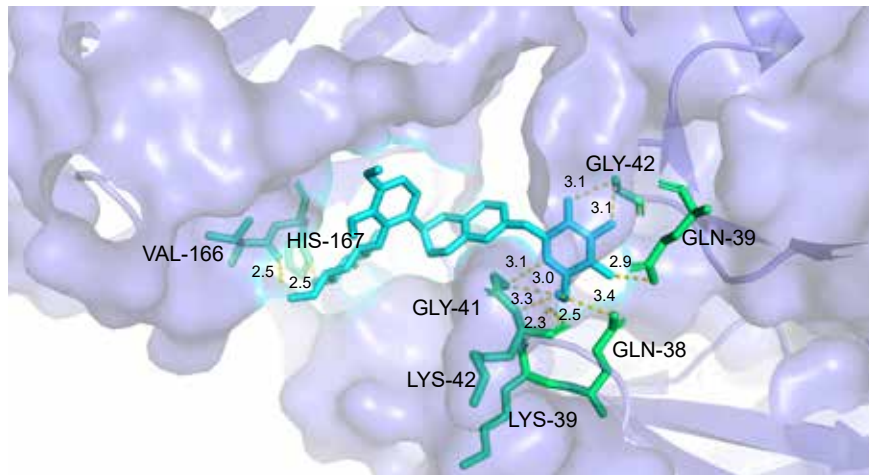

B

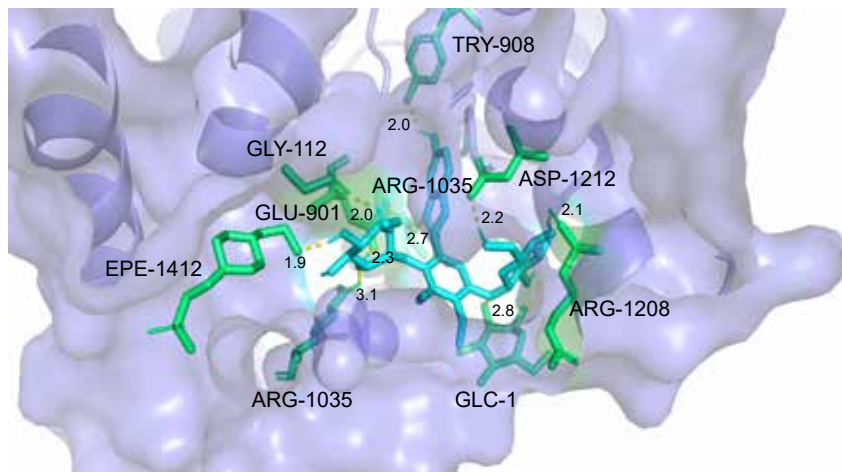

C

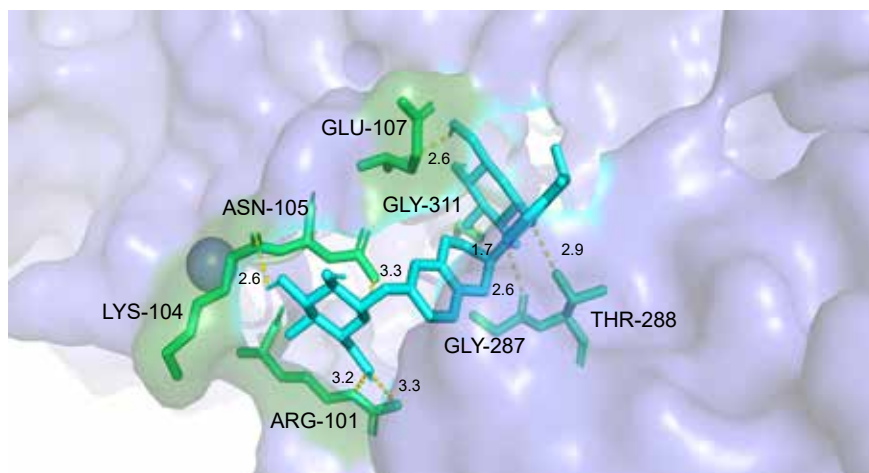

Supplement: Supplementary file 7 — Additional file 7: Figure S7. Molecular docking results. The amplification image of 3 prognostic genes with active ingredients. SPP1 and isomucronulatol 7-O-beta-glucosideADRB1 and 5’-hydroxyiso-muronulatol-2’,5’-di-O-glucosideADH1C and isomucronulatol 7-O-beta-glucoside. [file 12890_2023_2445_MOESM7_ESM.pdf]

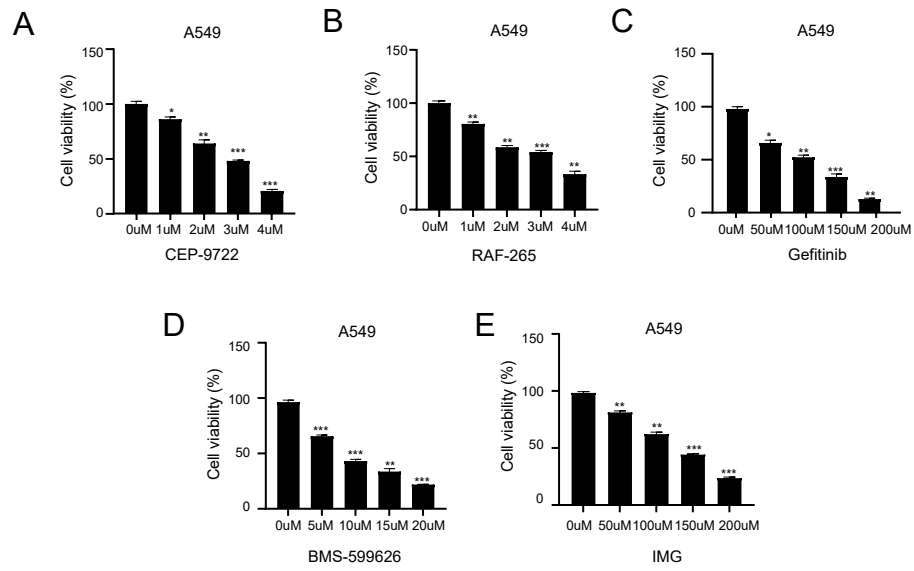

Supplement: Supplementary file 8 — Additional file 8: Figure S8. The CCK8 results of A549 cells withCEP-9722,RAF-265,Gefitinib,BMS-599626,IMG. [file 12890_2023_2445_MOESM8_ESM.pdf]
