# Supplementary material for: The combination therapy of isomucronulatol 7-O-beta-glucoside (IMG) and CEP-9722 targeting ferroptosis-related biomarkers in non-small cell lung cancer (NSCLC)
Source: BMC Pulm Med. 2023 May 11;23:162. doi: 10.1186/s12890-023-02445-0 (PMC10173508; doi:10.1186/s12890-023-02445-0)

^{1}H NMR (400 MHz, METHANOL-d_4) δ ppm 2.85 (dd, J=15.81, 4.02 Hz, 1 H) 3.00 (dd, J=15.81, 10.79 Hz, 1 H) 3.34 - 3.55 (m, 5 H) 3.71 (dd, J=12.05, 4.77 Hz, 1 H) 3.81 (s, 3 H) 3.80 (s, 3 H) 3.87 - 3.94 (m, 1 H) 4.00 (t, J=10.04 Hz, 1 H) 4.19 - 4.39 (m, 1 H) 4.86 (br. s., 1 H) 6.46 (d, J=8.53 Hz, 1 H) 6.55 (d, J=2.51 Hz, 1 H) 6.62 (dd, J=8.28, 2.51 Hz, 1 H) 6.78 (d, J=8.78 Hz, 1 H) 6.98 (d, J=8.28 Hz, 1 H)

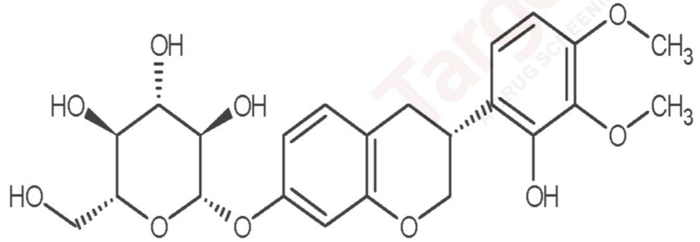

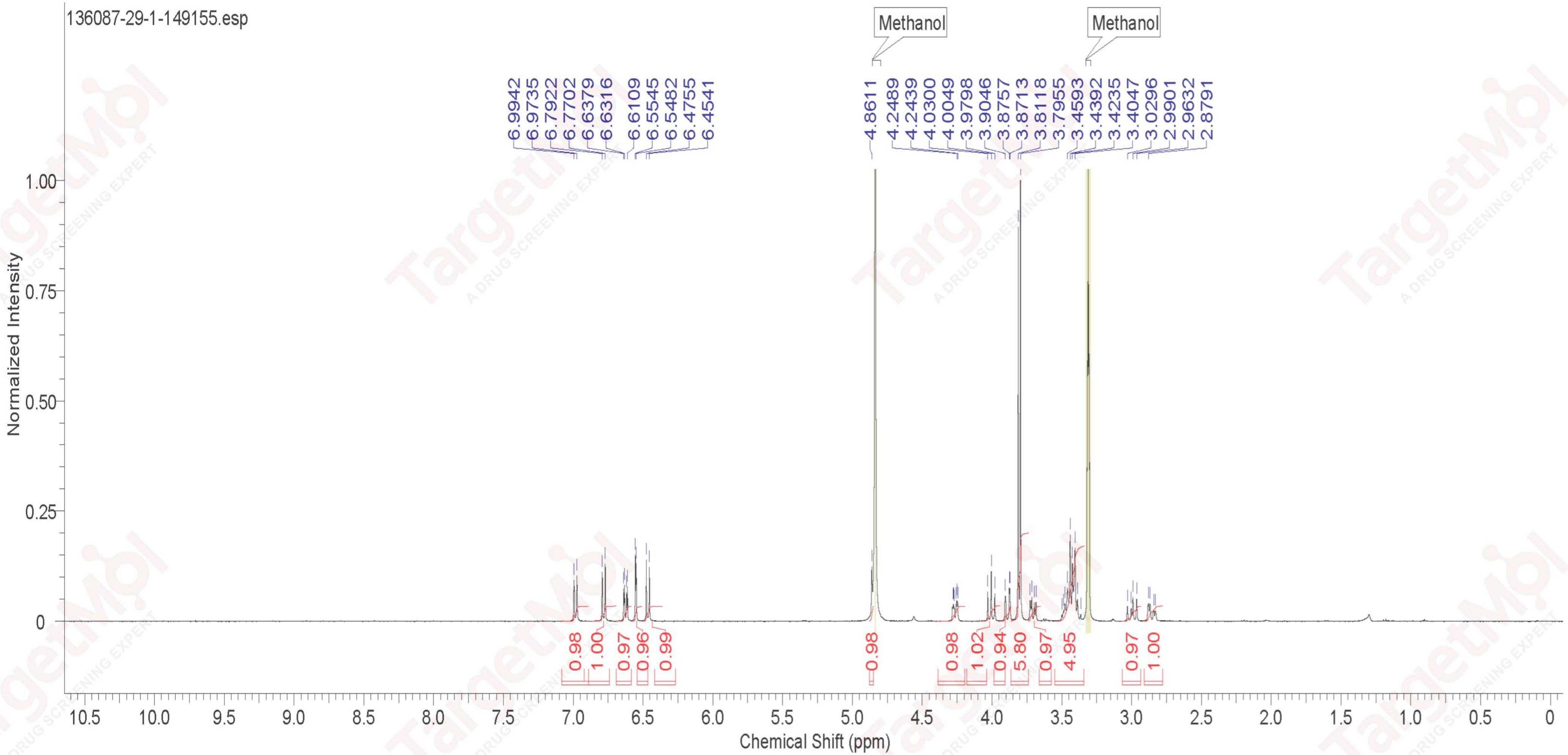

Supplement: Supplementary file 14 — Additional file 14. The IMG HNMR report from TargetMOI. [file 12890_2023_2445_MOESM14_ESM.pdf]
